# Gut microbiota changes after metabolic surgery in adult diabetic patients with mild obesity: a randomised controlled trial

**DOI:** 10.1186/s13098-021-00672-1

**Published:** 2021-05-21

**Authors:** Eva Lau, Eugeni Belda, Paul Picq, Davide Carvalho, Manuel Ferreira-Magalhães, Maria Manuel Silva, Isaac Barroso, Flora Correia, Cidália Pina Vaz, Isabel Miranda, Adelino Barbosa, Karine Clément, Joel Doré, Paula Freitas, Edi Prifti

**Affiliations:** 1 Department of Endocrinology and Nutrition, Centro Hospitalar de S. João, Alameda Professor Hernani Monteiro, 4200-319 Porto, Portugal; 2 CINTESIS - Center for Health Technologies and Information Systems Research - Faculty of Medicine, University of Porto, Porto, Portugal; 3 Integromics, Institute of Cardiometabolism and Nutrition, ICAN, Paris, France; 4 I3S – Instituto de Investigação e Inovação em Saúde, Faculty of Medicine, University of Porto, Porto, Portugal; 5 Health Information and Decision Sciences Department - Faculty of Medicine, Porto University, Porto, Portugal; 6 Department of Biochemistry, Centro Hospitalar de S. João, Porto, Portugal; 7 EpiUnit – Instituto de Saúde Pública, University of Porto, Porto, Portugal; 8 Department of Nutrition, Centro Hospitalar de S. João, Porto, Portugal; 9 Faculty of Nutrition and Food Science, Porto, Portugal; 10 Department of Pathology, Division of Microbiology, Faculty of Medicine, University of Porto, Porto, Portugal; 11 Surgery and Physiology, Cardiovascular Research Center, Faculty of Medicine, University of Porto, Porto, Portugal; 12 Department of Surgery, Centro Hospitalar de S. João, Porto, Portugal; 13 Sorbonne Université, INSERM, NutriOmics Research Unit, Pitié-Salpêtrière Hopital, Paris, France; 14 Université Paris-Saclay, INRA, MetaGenoPolis, AgroParisTech, MICALIS, 78350 Jouy-en-Josas, France; 15 Unité de Modélisation Mathématique et Informatique des Systèmes Complexes, IRD, Sorbonne Université, UMMISCO, Paris, France

**Keywords:** Diabetes mellitus, Insulin resistance, Microbiome, Roux-en-Y gastric bypass, Weight loss

## Abstract

**Background:**

Roux-en-Y gastric bypass (RYGB) surgery is one of the most efficient procedures for the treatment of obesity, also improving metabolic and inflammatory status, in patients with mild obesity. The underlying mechanisms have not been fully understood, but gut microbiota is hypothesized to play a key role. Our aim was to evaluate the association between gut microbiota changes and anthropometric, metabolic and inflammatory profiles after metabolic surgery compared with medical therapy, in type 2 diabetic (T2DM) adults with mild obesity (BMI 30–35 kg/m^2^).

**Methods:**

DM^2^ was an open-label, randomised controlled clinical trial (RCT: ISRCTN53984585) with 2 arms: (i) surgical, and (ii) medical. The main outcome was gut microbiota changes after: metabolic surgery (Roux-en-Y gastric bypass—RYGB) *versus* standard medical therapy. Secondary outcomes included anthropometric, metabolic and inflammatory profiles. Clinical visits, blood workup, and stool samples were collected at baseline and months (M)1, 3, 6, 12. Gut microbiota was profiled using 16S rRNA targeted sequencing.

**Results:**

Twenty patients were included: 10 in surgical and 10 in medical arm. Anthropometric and metabolic comparative analysis favoured RYGB over medical arm. At M12, the percentage of weight loss was 25.5 vs. 4.9% (*p* < 0.001) and HbA1c was 6.2 vs. 7.7% (*p* < 0.001) respectively. We observed a continuous increase of genus richness after RYGB up until M12. In the medical arm, genus richness ended-up being significantly lower at M12. Composition analysis indicated significant changes of the overall microbial ecosystem (permanova *p* = 0.004, [*R*^2^ = 0.17]) during the follow-up period after RYGB. There was a strong association between improvement of anthropometric/metabolic/inflammatory biomarkers and increase in microbial richness and Proteobacterial lineages.

**Conclusions:**

This was the first RCT studying composite clinical, analytic, and microbiome changes in T2DM patients with class 1 obesity after RYGB *versus* standard medical therapy. The remarkable phenotypic improvement after surgery occurred concomitantly with changes in the gut microbiome, but at a lower level.

*Trial registration:* ISRCTN53984585

**Supplementary Information:**

The online version contains supplementary material available at 10.1186/s13098-021-00672-1.

## Background

Obesity and type 2 diabetes mellitus (T2D) are both metabolic diseases that have expanded worldwide, reaching epidemic proportions and increasing patients’ morbidity and mortality [1]. Close interaction and imbalance between epigenetic and environmental factors are the primers for the complex etiological pathways of both diseases. Recent studies have emphasised the role of gut microbiota in metabolic regulation—specifically in energetic storage dysfunction and systemic inflammation [2, 3].

The “Microbiome Hypothesis” is based on the fact that humans host 10^14^ bacteria in the gut, which perform a variety of physiological functions, ranging from protective to metabolic regulation, including carrying out an active part in glucose and lipid metabolism [4, 5]. Intestinal dysbiosis, characterised by changes in gut microbiota composition, has been shown to play an essential role in obesity and T2D [6, 7]. Ley et al*.* showed a shift towards a higher relative abundance of *Bacteroidetes* and a decreased quantity of *Firmicutes* in patients with obesity who lost weight through low-calorie diets [8]. Furthermore, experimental data has highlighted the role of gut microbiota in nutrient absorption, as well as in the maintenance of gut barrier integrity and lipogenesis and hormonal status, leading to an increasing interest in shaping human gut microbiota composition in order to prevent and treat obesity and restore glucose homeostasis [9, 10].

Recent clinical trials reported that bariatric surgery, namely Roux-en-Y gastric bypass (RYGB) or bilio-pancreatic diversion, was the most effective treatment for combined weight loss and improvement/remission of T2D in patients with severe obesity [body mass index (BMI) >  = 35 kg/m^2^] [11, 12]. Such encouraging results led to further research adopting the same approach in patients with mild obesity (BMI 30–35 kg/m^2^) and the outcomes also proved the efficacy of this surgery in this subgroup of patients with obesity [13]. The mechanism by which T2D improves rapidly after RYGB, before significant weight loss, has not yet been completely understood, but recent studies have shown that gut microbiota modulation contributes to beneficial metabolic effects [14, 15]. Furet et al. demonstrated a shift toward an increase of *Faecalibacterium prausnitzii* in diabetic patients which was associated with a reduction in low-grade inflammatory state in obesity and diabetes [14].

To our knowledge, no randomised controlled clinical studies have been carried out assessing the association between gut microbiota changes and metabolic outcomes after RYGB in diabetic patients with mild obesity, compared to standard medical therapy. The main aim of our study was to evaluate gut microbiota changes after metabolic surgery *versus* standard medical therapy in diabetic adult patients with class 1 obesity. Secondary aims included: (1) the assessment of anthropometric, metabolic and inflammatory changes after interventions; and (2) the study of associations between gut microbiota alterations with anthropometric, metabolic, and inflammatory changes. This research contributes to improve our understanding of the intricate role of the gut microbiota in metabolic regulation.

## Methods

### Study design and participants

The Diabetes, Microbiota and Metabolic surgery (DM^2^) study was an open-label, randomised controlled clinical trial (RCT) carried out at the Centro Hospitalar São João (CHSJ) in Porto, Portugal, which was designed to assess gut microbiota changes and T2D resolution in patients with mild obesity after metabolic surgery *versus* standard medical therapy. The Ethics Committee from CHSJ approved the clinical protocol (ref 116/13) and all patients provided written informed consent. This RCT was registered in International Standard Randomised Controlled Trial Number Registry (ISRCTN), with the number ISRCTN53984585 (http://www.isrctn.com/ISRCTN53984585).

We used electronic medical records to identify candidates to participate in the trial, and between May 2014 and August 2014 we screened 42 patients at the CHSJ Endocrinology outpatient centre. Assuming rates of diabetes resolution to be 83%, with a 20% dropout rate in each study arm [12], required an enrolment of 10 patients in each category. This provided a power of > 90% to detect differences between the 2 groups, using a 2-sided alpha of 0.05. Consequently, we included 20 participants who were randomly assigned to one of the two study arms, with a 1:1 ratio, using a computer-generated randomisation procedure, to receive either RYGB or standard medical therapy. Blinding was unsuitable for use, due to the major differences between the two treatment therapies; however, participants and researchers were only aware of the study arm of each participant after patients’ informed consent and the random allocation concealment.

Inclusion criteria were as follows: age ≥ 20 and ≤ 65 years old; BMI ≥ 30 and < 35 kg/m^2^; previous diagnosis of T2D, according to the American Diabetes Association (ADA) definition, and under antidiabetic medical therapy [16]; duration of diabetes > 3 months; overnight-fasting C-peptide > 0.7 ng/ml; negative anti-GAD autoantibody; possible eligibility for general anesthesia; ability and willingness to participate in the study, with an understanding of the requirements of each arm of the study (written informed consent). The exclusion criteria were: specific contraindication to obesity surgery; diabetes secondary to a specific disease (maturity-onset diabetes of the young, latent autoimmune diabetes in adult or pancreatitis); having taken any antibiotic, probiotic, or prebiotic agents during the month before randomisation; pregnancy; debilitating disease; any psychological condition which could hamper a patient’s cooperation; any condition which, in the opinion of the researcher, could have meant that participation in the study was risky or could have biased the results.

### Surgical arm: Roux-en-Y gastric bypass surgery

Participants in the surgical arm underwent RYGB—whereby a 30 ± 10 mL capacity subcardial gastric pouch was created on a nasogastric 36F calibrating tube by sectioning the stomach with a linear stapler 3–4 cm horizontally on the lesser curve, 4 cm distant from the e–g junction, and then vertically until attainment of the angle of Hiss. After identification of the Treitz ligament, the jejunum was transected at 100 cm from the ligament of Treitz and the two stumps were closed. The distal stump was anastomosed to the distal end of the gastric pouch. Finally, the proximal stump of the transacted bowel was joined end-to-side to the jejunum 150 cm distant from the gastroenterostomy.

### Medical arm: Standard medical therapy

Participants in the medical arm were managed with antidiabetic standard medical therapy. This was defined as the use of lifestyle (nutrition and exercise counselling) guidelines by ADA to optimise weight loss and glycaemic control, along with frequent glucose self-monitoring and titration strategies, and also drug therapy for hyperglycaemia and restoration of pancreatic cells function [16]. In addition, all subjects were treated according to ADA guidelines for lipid and blood pressure targets.

### Follow-up

The follow-up period ran from October 2014 to May 2016. Clinical visits and laboratory tests were carried out at six points in time: (i) during the screening period; (ii) at baseline, after randomisation (M0); (iii) and at four moments of follow-up—(Months M1; M3; M6; M12 after the RYGB in surgical arm, or after the first appointment in medical arm).

Clinical visits were carried out at all six moments of assessment. Patients were evaluated by an endocrinologist who performed a complete medical history and physical examination, including anthropometric measurements (body weight, height, waist circumference, visceral fat area, body fat mass and fat-free mass) using bio-impedance analysis (Inbody®, model 720). Dietary records were also evaluated by a nutritionist, who carried out a detailed dietary intake (quality and quantity) for the 24 h period before the interview. Patients also maintained dietary records for the 72 h period before the clinical visit. Laboratory tests included blood biochemical tests and gut microbiota analysis. Blood biochemical tests were conducted at all six moments of assessment and patients had at least eight hours of fasting, with trained nurses collecting blood samples at the beginning of the clinical visit. Gut microbiota analyses were conducted at M0, M1, M3, M6 and M12, and faecal samples were collected by participants at home, after appropriate exemplification and training of the collection procedure. Faecal samples were collected in the morning, before breakfast. Whole stools were self-collected in sterile boxes and stored at − 20 °C. Samples were treated in the laboratory and stored as 200 mg aliquots at − 80 °C until further analysis.

### Biochemical tests

Routine chemical analyses were evaluated using an enzymatic colorimetric test using the Olympus AU 5400 clinical chemistry analyser (Beckman Coulter®, USA), including glucose (fasting glucose, insulinemia and C-peptide) and lipid profile components of the serum [total cholesterol (TC), high-density lipoprotein cholesterol (HDL-C), and Triglycerides (TG)]. Low-density lipoprotein cholesterol (LDL-C) was calculated according to the Friedewald Eq. [17]. The measurement of hemoglobin A_1C_ (A1c) was performed using ion-exchange high-performance liquid chromatography (HPLC) with a Variant™ Turbo A1c (Bio-Rad®, USA). Serum high-sensitivity C-Reactive Protein (hsCRP) was assessed using particle-enhanced immunonephelometric tests with a BN™ II laser nephelometer (Siemens®, Erlangen, Germany). Insulin was assayed using an electrochemiluminescence immunotest with a Cobas e411 automated analyser (Roche®, Germany). C-Peptide was assessed using electrochemiluminescence immunoassay with a Cobas e411 automated analyser (Roche®, Germany).

### Gut microbiota data processing

Gut microbiota profiling was carried out using 16S rRNA targeted sequencing of the V3-V4 hypervariable region using primers derived from Klindwordth et-al. [18], using a methodological approach developed by GenoScreen^©^ (France). This process consists of three steps: (1) the preparation of libraries of amplicons according to the Metabiote® tool, limiting the bias of amplification between samples and including a positive control (the artificial bacterial community "ABC control"), with a first negative control (background of the whole process of libraries construction) and a second negative control (background of the stool extraction step); (2) sequencing of the 16S amplicons libraries on an Illumina MiSeq "paired-end" 2 × 250 bp run; and (3) sorting by sample indexes and reassembly of the two "paired-end" reads to obtain full length 16S rDNA sequences. The 16S library constructions were carried out using 5 ng of gDNA, extracted according to the Metabiote® protocol developed by GenoScreen^©^.

The raw demultiplexed sequences were processed using *mothur*, with default parameters. Abundance tables at the genus level were rarefied at 2059 reads and were normalised before statistical analyses, using the total count procedure. The Bray–Curtis beta-diversity distance matrix was computed using the *vegdist* function of a vegan R package from the rarefied genus abundance matrix [19]. Principal Coordinate Analysis (PCoA) from Bray–Curtis beta-diversity matrix was carried out with the *cmdscale* function of a vegan R package.

Enterotype classification was carried out following the Dirichlet Multinomial Mixture (DMM) method of Holmes et al. [20], using the rarefied genus abundance matrix as the input. The DMM approach groups samples together if their taxon abundances can be modelled by the same Dirichlet-Multinomial (DM) distribution. Genus richness was estimated using different methods. Rarefaction to 6,000 reads per sample and upsizing procedure were used to estimate genus diversity. This consisted in a 10-times rarefaction procedure, with different alpha diversity metrics (observed genera, Chao1, ACE as richness estimators; Shannon, Simpson and InvSimpson as evenness estimators). We observed differences in sequencing depth between samples from each group, however this variability was omitted after the rarefaction procedure.

### Outcomes

The main outcome of the DM^2^ study was the quantification of gut microbiome diversity and composition after RYGB, compared with standard medical therapy. The secondary outcomes included the relation between T2D remission/improvement (as measured by anthropometric, metabolic and inflammatory biomarkers) and gut microbiota composition and modification throughout the intervention. Both outcomes were assessed at M0, M1, M3, M6 and M12. Diabetes remission was defined as A1c < 6.5%, without using any antidiabetic medication. Diabetes improvement was considered if patients still required non-insulin antidiabetic drugs, but at lower doses compared to the baseline (without insulin use), as well as A1c levels ≤ 7.0% [13].

### Statistical analysis

All values are described as mean and standard deviation (SD). Homeostasis model assessment of insulin resistance (HOMA-IR), percentage of total weight lost (%WL) and BMI were calculated, in accordance with the literature. The composition of microbiota was expressed with the mean of the normalised relative abundance values. PCoA transformation of the multidimensional data at the genus level was carried out, using the Bray–Curtis beta-diversity distance matrix. A PERMANOVA test on *vegan* R package with n = 999 permutations was carried out to test for differences in microbiome composition after surgery and interventions for the medical arm. Statistical analyses of the microbiome were carried out using the *momr* and *relome* R packages developed at Institute of Cardiometabolism and Nutrition (ICAN), France. Non-parametric tests (Wilcoxon, Kruskal-Willis or Spearman correlations) were performed when analyzing microbiome data. Benjamini–Hochberg multiple testing adjustment was applied (FDR < 0.1 indicates significance).

Paired Mann–Whitney tests were performed to analyse the changes in these parameters between baseline and the various points in time, for each study arm category. Linear regression was used to analyse associations between continuous variables in different timepoints, for each study arm, and *R*^2^ effect size was also calculated. All inferential statistical computations were considered to be significant when *p*-values were < 0.05. Statistical analysis was carried out using SPSS version 25 (SPSS IBM, New York, NY, USA) and R.

## Results

### Clinical and biological baseline characteristics

**Fig. 1:**
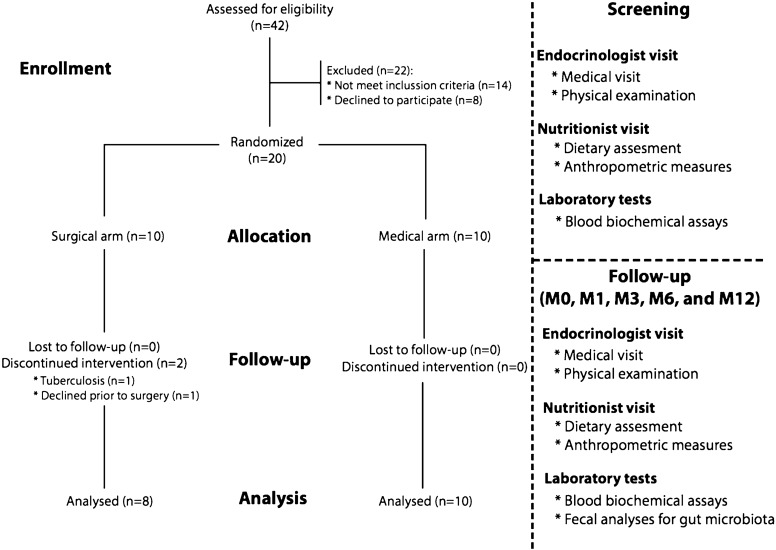
Study flowchart. *M* month

We screened 42 patients, from which we randomised 20 participants in the DM^2^ study (see Fig. 1). There were two dropouts in the surgical arm: one patient quit the study after allocation and the other was excluded after surgery, due to newly-diagnosed tuberculosis.

Patient’s characteristics are described in Table 1. The average age was 53 vs. 58 years old, BMI was 33.6 vs. 32.0 kg/m^2^, A1c was 8.7 vs. 8.2%, and women were 50% *vs*. 30%, for the surgical arm *vs*. medical arm, respectively. There were no significant differences in BMI, A1c, fasting glucose, insulin, C-peptide levels, or HOMA-IR between the two arms. However, patients who underwent RYGB had significantly higher weight, visceral fat area, body fat mass, and serum hsCRP at baseline (Table 1).

**Table 1:** Clinical and biological characteristics at all time points in surgical arm *versus* medical arm

	Baseline				Month 1				Month 3				Month 6				Month 12			
	Surgical arm N=8	Medical arm N=10	*p*	*q*	Surgical arm N=8	Medical arm N=10	*p*	*q*	Surgical arm N=8	Medical arm N=10	*p*	*q*	Surgical arm N=8	Medical arm N=10	*p*	*q*	Surgical arm N=8	Medical arm N=10	*p*	*q*
Age (years)	53.4 (8.78)	58.2 (4.73)	0.191	0.48	–	–	–	–	–	–	–	–	–	–	–	–	–	–	–	–
Female (%)	4 (50)	3 (30)	0.630	–	–	–	–	–	–	–	–	–	–	–	–	–	–	–	–	–
Diabetes duration (years)	8.12 (4.73)	10.0 (4.42)	0.404	0.620	–	–	–	–	–	–	–	–	–	–	–	–	–	–	–	–
Weight (Kg)	92.5 (7.65)	83.9 (9.10)	**0.046**	0.220	82.6 (4.44)	81.2 (8.82)	0.677	0.860	76.0 (5.77)	79.3 (8.56)	0.358	0.570	71.0 (8.64)	79.5 (8.93)	0.058	0.084	69.2 (11.8)	79.7 (9.07)	0.058	0.096
BMI (%)	33.6 (1.84)	32.0 (1.56)	0.069	0.230	30.9 (3.74)	31.1 (1.78)	0.946	0.950	27.7 (1.36)	30.3 (2.07)	**0.005**	**0.048**	25.7 (1.51)	30.4 (2.38)	**<0.001**	**0.0012**	24.6 (2.87)	30.5 (2.55)	**<0.001**	**0.0023**
%WL (%)	–	–	–	–	10.5 (2.82)	3.15 (2.51)	**<0.001**	**<0.001**	17.7 (2.96)	5.49 (3.27)	**<0.001**	**<0.001**	23.4 (4.62)	5.18 (4.28)	**<0.001**	**<0.001**	25.5 (8.56)	4.88 (5.53)	**<0.001**	**0.0018**
Waist circumference (cm)	113 (4.01)	108 (5.47)	0.055	0.220	104 (4.74)	104 (4.48)	0.845	0.890	97.9 (5.59)	103 (4.29)	**0.043**	**0.160**	93.1 (4.21)	101 (4.93)	**0.003**	**0.0098**	88.2 (5.99)	101 (5.64)	**<0.001**	**0.0023**
Visceral fat area (cm²)	157 (15.3)	126 (16.7)	**0.001**	**0.0087**	139 (17.2)	119 (16.5)	**0.022**	**0.160**	116 (19.9)	115 (17.2)	0.897	0.930	91.4 (26.0)	117 (19.8)	**0.037**	**0.061**	84.0 (24.0)	117 (18.8)	**0.007**	**0.0190**
Body fat mass (Kg)	39.9 (4.60)	30.2 (3.89)	**<0.001**	**0.0064**	33.6 (5.96)	28.1 (4.19)	**0.049**	**0.190**	26.4 (5.84)	26.9 (4.06)	0.838	0.930	19.9 (6.31)	28.2 (4.02)	**0.007**	**0.020**	17.9 (5.71)	27.4 (4.71)	**0.002**	**0.0086**
Fat-free mass (Kg)	52.5 (9.69)	53.7 (9.48)	0.802	0.860	49.0 (8.80)	53.1 (9.32)	0.356	0.600	49.7 (9.10)	52.4 (8.47)	0.529	0.630	51.1 (10.5)	52.2 (8.80)	0.821	0.920	51.2 (9.60)	112 (193)	0.343	0.380
Fasting glucose (mg/dL)	175 (27.9)	164 (43.6)	0.528	0.750	154 (50.4)	144 (41.0)	0.662	0.860	128 (21.1)	144 (33.6)	0.244	0.460	117 (16.5)	159 (22.6)	**<0.001**	**0.0024**	124 (20.6)	170 (38.7)	**0.007**	**0.019**
HbA_1c_ (%)	8.69 (1.03)	8.19 (0.56)	0.246	0.490	7.57 (0.78)	7.45 (0.66)	0.722	0.860	6.74 (0.71)	7.05 (1.14)	0.487	0.620	6.24 (0.66)	7.27 (1.14)	**0.030**	**0.056**	6.17 (0.68)	7.74 (0.69)	**<0.001**	**0.0021**
Insulin (uU/mL)	13.7 (9.45)	16.3 (11.0)	0.602	0.800	6.59 (2.28)	18.5 (14.0)	**0.026**	**0.160**	6.03 (1.58)	17.2 (12.4)	**0.019**	**0.091**	5.67 (3.28)	18.7 (10.4)	**0.003**	**0.010**	4.72 (2.09)	18.9 (16.5)	**0.024**	**0.048**
C-peptide (ng/mL)	2.54 (1.27)	3.43 (1.80)	0.239	0.490	2.34 (0.66)	3.71 (1.98)	0.064	0.20	2.04 (0.45)	2.96 (1.38)	0.072	0.230	1.82 (0.55)	3.08 (1.20)	**0.011**	**0.024**	1.70 (0.59)	3.19 (1.60)	**0.019**	**0.042**
HOMA-IR	5.99 (4.38)	6.58 (4.27)	0.780	0.860	2.54 (1.12)	6.52 (5.01)	**0.035**	**0.170**	1.90 (0.53)	6.11 (4.31)	**0.013**	**0.082**	1.59 (0.81)	7.20 (3.66)	**0.001**	**0.0039**	1.43 (0.66)	8.60 (8.60)	**0.027**	**0.050**
Total cholesterol (mg/dL)	170 (36.0)	165 (56.1)	0.821	0.860	160 (33.5)	153 (42.1)	0.712	0.860	167 (31.1)	146 (53.9)	0.309	0.530	162 (28.2)	160 (41.5)	0.874	0.920	173 (26.3)	155 (41.4)	0.286	0.370
Triglycerides(mg/dL)	124 (47.7)	186 (175)	0.308	0.560	114 (49.0)	182 (151)	0.210	0.570	108 (32.4)	176 (116)	0.106	0.230	91.9 (30.6)	144 (62.6)	**0.038**	**0.061**	100 (42.2)	169 (124)	0.131	0.190
HDL-c (mg/dL)	52.0 (15.9)	39.7 (11.8)	0.093	0.270	47.4 (10.9)	40.5 (14.9)	0.275	0.570	51.0 (12.2)	40.0 (13.2)	0.086	0.230	54.4 (7.89)	40.7 (12.0)	**0.011**	**0.024**	62.9 (9.64)	41.5 (15.5)	**0.003**	**0.0088**
LDL-c(mg/dL)	93.4 (23.1)	97.9 (48.7)	0.799	0.860	90.9 (27.1)	87.1 (33.6)	0.795	0.890	94.5 (25.0)	70.6 (34.3)	0.107	0.230	89.6 (20.8)	90.4 (32.7)	0.952	0.950	89.8 (19.8)	79.9 (32.6)	0.441	0.440
Systolic BP (mmHg)	132 (11.6)	132 (10.5)	1.000	1.000	117 (9.11)	121 (8.71)	0.300	0.570	121 (10.3)	117 (14.7)	0.453	0.610	119 (10.1)	124 (8.29)	0.256	0.350	120 (10.4)	124 (8.56)	0.311	0.370
Diastolic BP (mmHg)	70.6 (4.84)	66.8 (11.8)	0.370	0.620	62.8 (4.17)	66.7 (12.7)	0.376	0.60	65.6 (5.90)	65.9 (7.46)	0.932	0.930	65.6 (4.37)	68.2 (8.69)	0.428	0.540	63.2 (5.97)	67.0 (8.65)	0.294	0.370
hsCRP (mg/dL)	6.89 (2.95)	2.61 (1.50)	**0.004**	**0.027**	6.25 (10.8)	1.36 (0.89)	0.242	0.570	1.94 (1.35)	1.43 (1.21)	0.421	0.610	1.12 (0.82)	1.30 (0.93)	0.677	0.800	0.82 (0.61)	1.39 (0.84)	0.118	0.180

*BMI* body mass index, *%WL* percentage of weight lost, *HbA*_*1c*_ Glycated hemoglobin, *HOMA-IR* homeostatic model assessment for insulin resistance; *HDL-c* high density lipoprotein cholesterol, *LDL-c* low density lipoprotein cholesterol, *BP* blood pressure, *hsCRP* high sensitivity C-reactive protein, *n.a.* not applicable

Bold values are statistically significant. Values between parentheses are standard deviation of the respective mean value

### Bariatric surgery outperforms medical therapy in improving patients’ anthropometric, metabolic and inflammatory profiles

One month after the beginning of the study, there was no evidence of differences in BMI between arms, however, %WL was significantly higher in the surgical category (10.5* vs.* 3.1%, *p* < 0.001; Table 1). Neither were any significant differences observed at this time in fasting glucose, A1c, or C-peptide between arms. Nonetheless, we found lower insulin levels (6.6 vs. 18.5 mg/dL, *p* = 0.026) and HOMA-IR (2.5 vs. 6.5, *p* = 0.035) in the surgical arm.

At three months of follow-up, differences in %WL were even more pronounced (17.7 vs. 5.5%, *p* < 0.001), and significant differences in BMI (27.7 vs. 30.3 kg/m^2^, *p* = 0.007) and waist circumference (97.9 vs. 103.3 cm, *p* = 0.034) were observed, whereas none were for fasting glucose, A1c, or C-peptide between both arms. Nevertheless, differences between arms increased as far as insulin levels (6.0 vs. 17.2 mg/dL, *p* = 0.019) and HOMA-IR (1.9 *vs*. 6.1, *p* = 0.013) were concerned.

At six months of follow-up, differences in BMI and %WL continued to be even greater (BMI: 25.7 vs. 30.4 kg/m^2^, *p* < 0.001; %WL: 23.4 vs. 5.2%, *p* < 0.001) and body fat measures were significantly lower in the surgical arm (visceral fat area, p = 0.029; and body fat mass, *p* = 0.003). Measures of insulin resistance were also significantly reduced in the surgical arm (A1c: 6.2 vs. 7.3%, *p* = 0.038; HOMA-IR: 1.6 vs. 7.2, p < 0.001), as were triglycerides (91.9 vs. 143.6 mg/dL, *p* = 0.049), while HDL-C was significantly increased (54.4 vs. 40.7 mg/dL, *p* = 0.014).

**Fig. 2:**
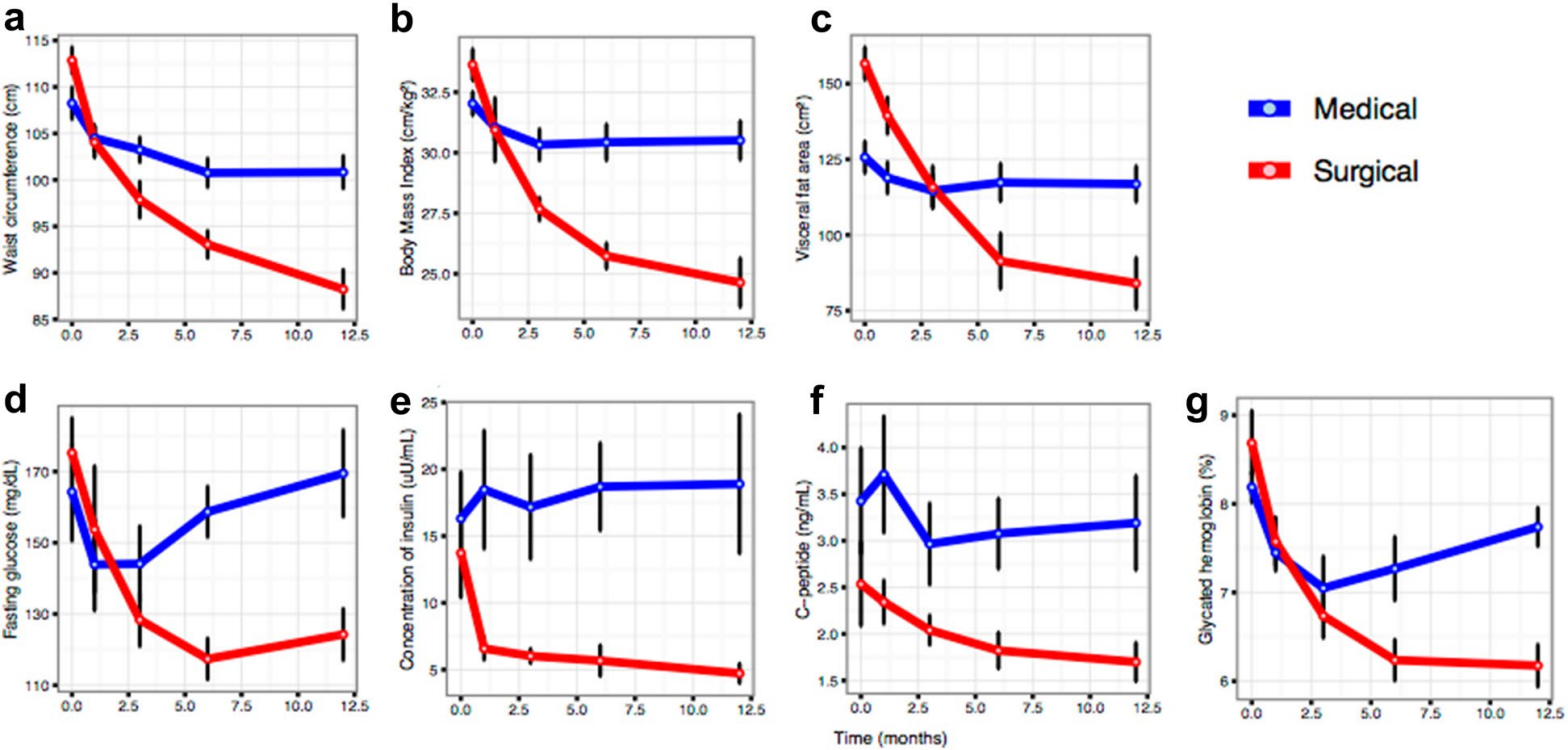
Clinical trajectories throughout the follow-up time points by study arms. Average values ± standard error values are depicted for each time point (baseline, 1, 3, 6 and 12 months). Blue and red colours indicate the medical and the surgical arm respectively. **a**–**c** corpulence measurements; **d**–**g** metabolic measurements

Lastly, at 12 months of follow-up, the average BMI was 24.6 vs. 30.5 kg/m^2^ (*p* < 0.001), and A1c was 6.2 vs. 7.7% (*p* < 0.001) in the surgical *vs*. medical arm, respectively (Table 1). The %WL was 25.5% in the surgical arm and 4.9% in the medical arm (*p* < 0.001). Waist circumference, visceral fat area and body fat mass were statistically lower in the surgical arm (*p* < 0.001, *p* = 0.007 and *p* = 0.002, respectively). In addition, fasting glucose, insulinemia, C-peptide, and HOMA-IR were significantly lower in the surgical arm (*p* = 0.007, *p* = 0.020, *p* = 0.020 and *p* = 0.027, respectively), and HDL-C was higher (*p* = 0.004). See Table 1 for absolute values for each parameter and Fig. 2 for summarised trajectories. At the final endpoint, all participants from the medical arm failed to achieve diabetes remission or improvement, however in the surgical arm, 5 participants (62.5%) experienced remission from their diabetes (*p* = 0.007 for comparison with medical arm, Chi^2^ test), 2 participants (25%) improved their phenotypes, and only 1 showed no improvement. Supplementary analyses were performed using the relative change between baseline and 12 months of follow-up. These results are shown in the supplementary material (Additional file 1: Table S1, Table S2 and Table S3, and Figure S1).

### Microbiota composition and clinical phenotypes before interventions

We hypothesised that the clinical changes that occurred in the study cohort may have been accompanied by changes in the gut microbiome. Therefore, we characterised the latter at different levels of specificity—such as richness, community type, and the genus taxonomic levels. In this study, we computed richness as being the number of present/observed genera. First, we observed no saturation at the genus level during the rarefaction analysis (Additional file 1: Figure S2A), which signifies that the sequencing depth may be insufficient to capture all of the complexity of the microbiome at the genus level. However, the limited sequencing depth would nevertheless allow the capture of the signal for the most prevalent and abundant rates. We found differences in sequencing depth between study arms (lower in surgical) (Additional file 1: Figure S2B). These differences were levelled out with a rarefaction procedure.

At baseline, we observed a lower genus richness (trend) in the surgical arm, compared with the medical arm (61.37 ± 9.16 vs 71.3 ± 11.19 respectively, p = 0.055) (Additional file 1: Figure S3A). Additionally, when correlating genus richness with clinical variables (Additional file 1: Table S4), we observed that the waist circumference (p = 0.014) and hsCRP inflammatory marker (*p* = 0.023) were negatively associated, as previously described [21, 22], (Additional file 1: Figure S3b–d).

Next, we studied the microbiome data using a community-based approach. Enterotypes were recognised as interesting describers of microbiome abundance data [23]. Indeed, recently an improved enterotyping approach, based on DMM has shown to be better for capturing a significant signal in the microbial communities [24]. We applied the DMM method, despite difficulties in determining the same optimal group number in the Laplacian profile—we fixed it for k = 4 for comparison of the results with the original study (Additional file 1: Figure S2c). The potential drivers of enterotype composition for different groups are: K1: Synergestes (2 genera), Desulfovibrio (delta-proteobacteria), Ruminococcaceae genera Clostridium IV, and Unclassified Ruminococcus; K2: Lachnospiraceae, Ruminococcus; K3: Actinobacteria (Bifidobacterium), Bacillales (even less abundant), Enterobacteriales (potentially pro-inflammatory). Additionally, decreased levels of the following were observed: *Faecalibacterium* (potentially anti-inflammatory) and Oscillibacteria (Ruminococcaceae); K4: Lachnospiraceae, *Roseburia*, and *Ruminococcus* (all Clostridiales) (data not shown).

These enterotype profiles are different from what is observed in previous studies [25–28]. Furthermore, no significant differences are seen in genus richness across enterotypes when considering the entire study cohort (data not shown) [24]. Contrary to what is expected, no significant associations are observed between enterotypes and clinical variables at baseline.

Next, we explored compositional changes between study arms at baseline by applying a PERMANOVA test (non-parametric MANOVA from Bray–Curtis beta diversity distance matrix computed with genus abundance data). No significant difference was observed (p = 0.095; Additional file 1: Figure S2d). When testing for differentially abundant features between surgical and medical arms at baseline, five genera were observed (not resisting multiple testing adjustment; Additional file 1: Figure S4).

### Microbiota changes during the intervention

**Fig. 3:**
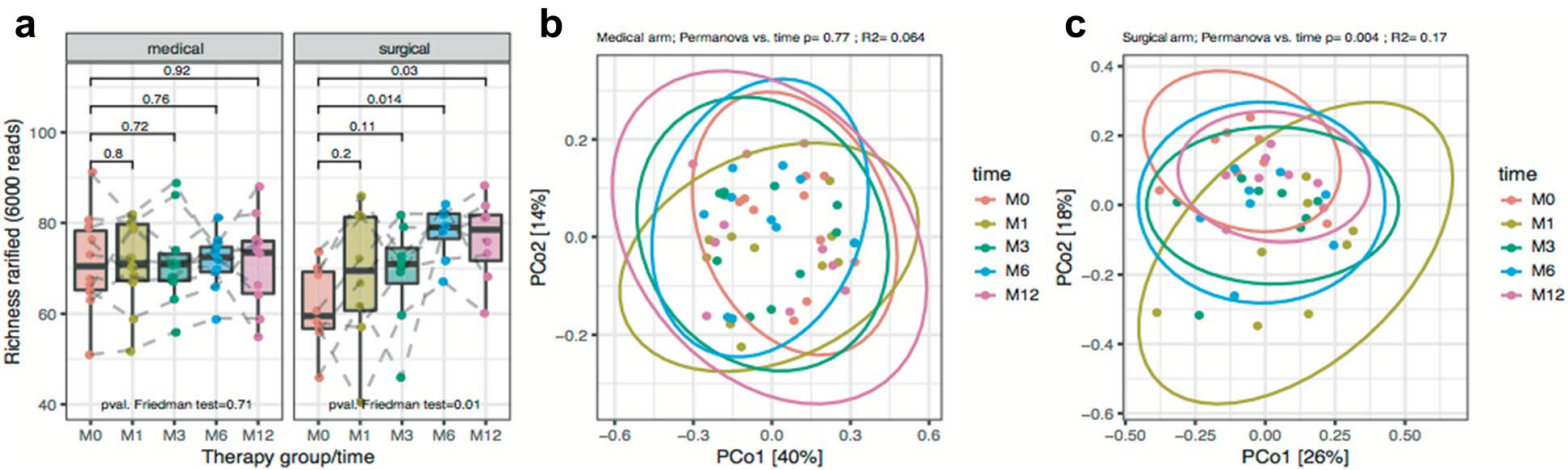
Gut genus richness throughout the follow-up time points and arms. **a** genus richness distribution across time in each study arm. **b** PCoA analysis of microbiome changes through time in the medical arm. **c** PCoA analysis of microbiome changes through time in the surgical arm. PERMANOVA tests were carried out over beta-diversity distance matrices computed from rarified genus abundance data testing for differences between time points in each study arm (*R*^2^ and *P* values shown)

Despite lower baseline genus richness in the surgical arm (61 ± 9 prokaryotic genera), we found a significant continuous increase until attaining 76 ± 9 prokaryotic genera at M12 (*p* = 0.03 on paired Wilcoxon tests). On the contrary, genus richness did not change throughout the intervention in the medical arm (71 ± 11 at baseline; 71 ± 10 at M12; *p* = 0.92 on paired Wilcoxon tests) (Fig. 3a). These findings confirm previous results in patients with severe obesity where microbial gene richness increases after RYGB [29].

The enterotype analyses did not allow us to observe any significant change throughout the intervention, which is probably a consequence of the small number of patients and stratification in the statistical tests. In the surgery arm, the composition analysis indicated significant changes in the microbiome (*R*^2^ = 0.17; permanova p = 0.004) (Fig. 3c). Changes in the medical arm were not significant (Fig. 3b), which confirmed the observations at the richness level.

**Fig. 4:**
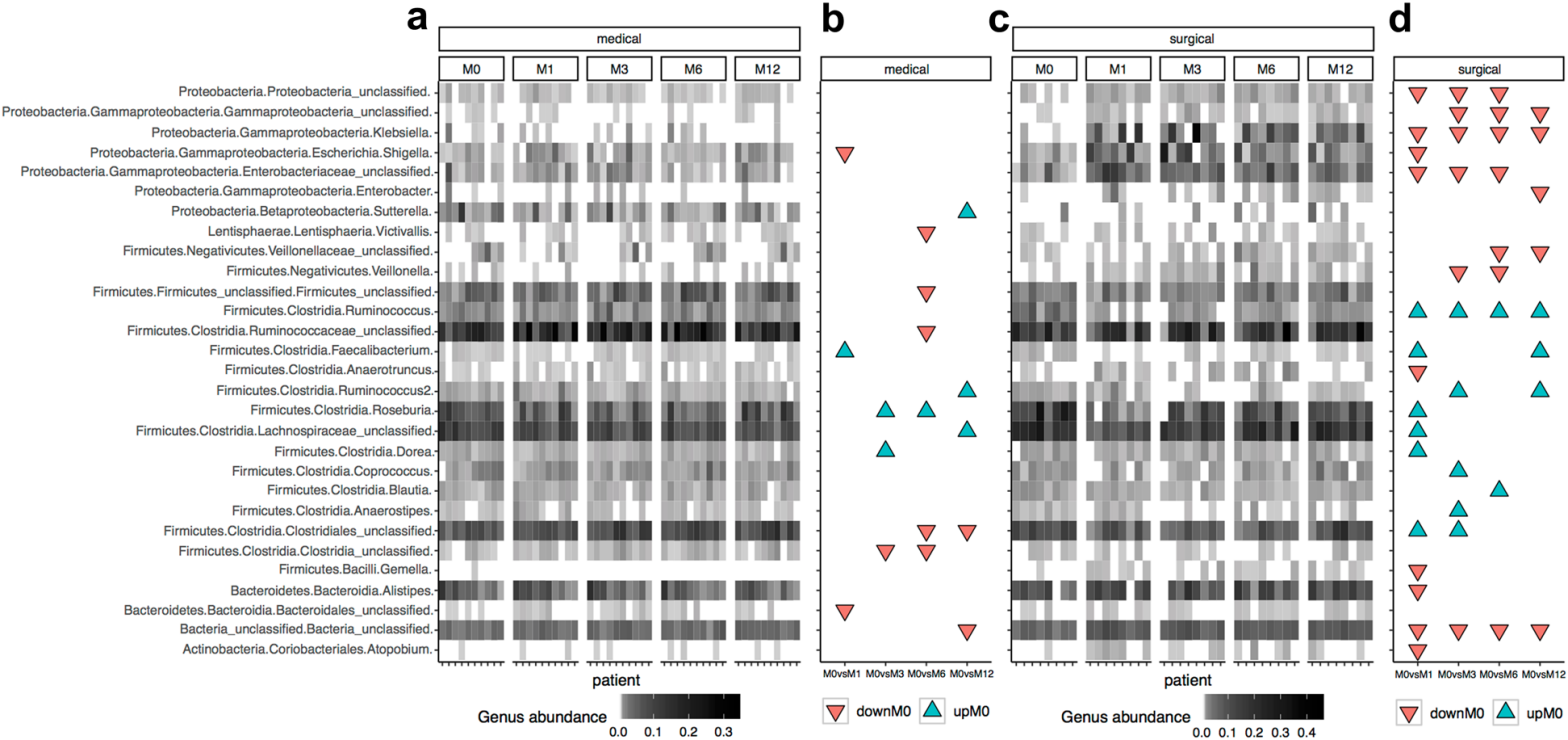
Abundance profiles of 29 bacterial genus with significant changes throughout the follow-up time points and arms. **a** Heatmap of relative abundances of these 29 genus features in patients of the medical arm. **b** Significant associations these 29 genus features between baseline and different study points in medical arm (DownM0 = Significant increases in genus abundance at different study points in comparison with baseline; UpM0 = Significant decreases in genus abundance at different study points in comparison with baseline; *P* value < 0.05, Paried Wicoxon rank-sum test). **c** and **d** panels are equivalent to panels **a** and **b** for patients of the surgery arm

Furthermore, we searched for specific genera that changed in abundance at the study endpoint, compared to baseline. We observed that *Ruminococcus*, *unclassified*_*Lachnospiraceae_* family and *Faecalibacterium* significantly decreased, while *Klebsiella*, *Gammaproteobacteria*, *Enterobacter*, *unclassified_Gammaproteobacteria*, *unclassified_Veillonellaceae* increased after 12 months of RYGB. In the medical arm, *unclassified_Lachnospiraceae* and *Sutterella* significantly decreased, while *unclassified*_*Clostridiales* and *unclassified*_*Bacteria* increased, when comparing baseline with M12 (Additional file 1: Figure S5). None of these changes did resist multiple testing adjustment though (Additional file 1: Table S5). The main changes in genus abundance comparing each timepoint of the study is represented in Fig. 4.

Finally, we explored the differences in the Firmicutes/Bacteroides ratio (rFB) during the interventions’ follow-up. This score is largely used in microbiome studies as a marker of ecosystem health. No significant differences between the two categories or through time were found in this case (Additional file 1: Figure S6a). However, in agreement with previous findings [30], the Proteobacteria/Firmicutes ratio (rPF) significantly increased after RYGB, but not in the medical arm (Additional file 1: Figure S6b).

### How do microbiome changes relate to phenotypic evolution?

**Fig. 5:**
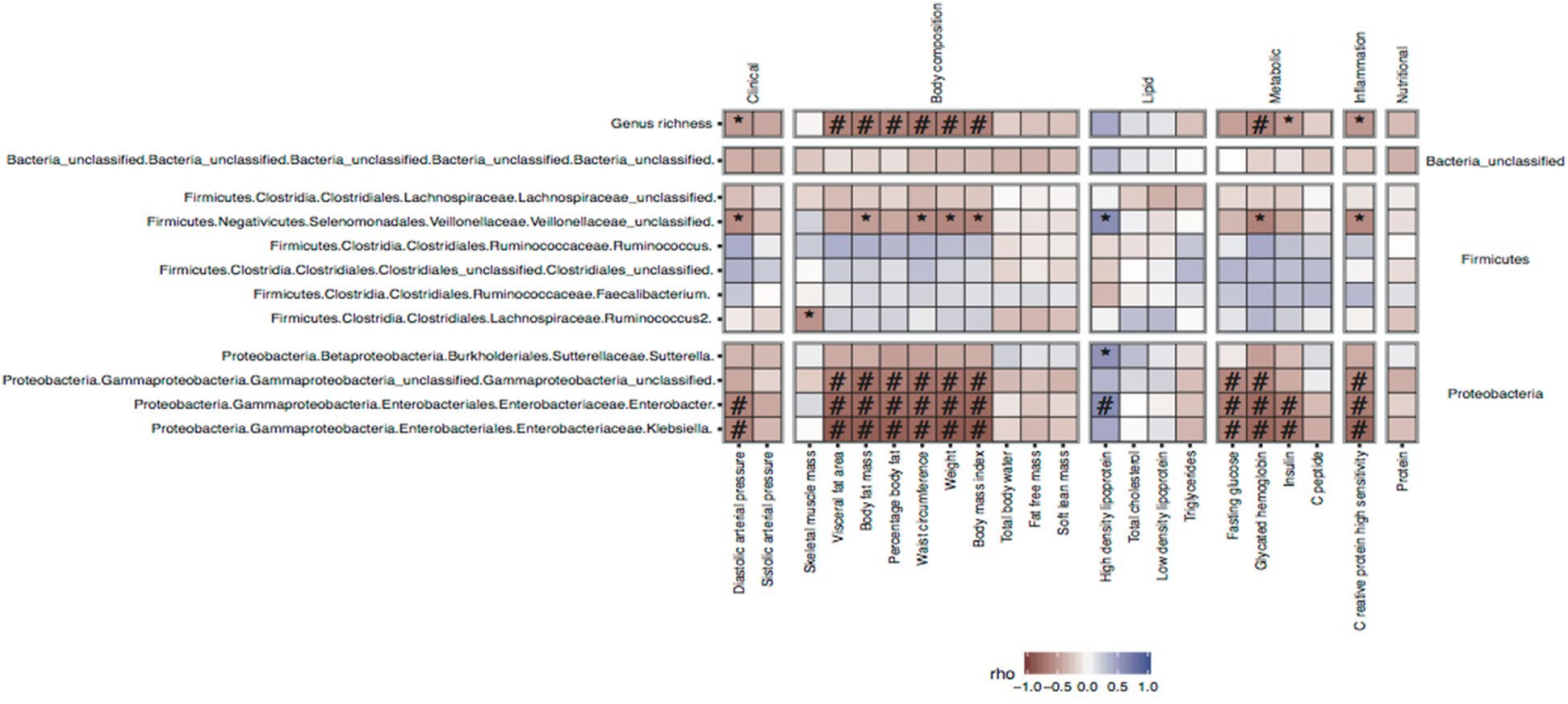
Heatmap of associations of changes between phenotypes and genera between baseline and 12 months of follow-up. Heatmap of spearman correlation coefficients between relative change associations of genus richness and target genera with changes in clinical variables. */^#^: *p*/*q* values < 0.05 in spearman correlation tests

We investigated the associations between relative changes in metabolic parameters and microbial richness. There was a strong inverse relationship between changes in body composition and anthropometric/metabolic markers (waist circumference, diastolic blood pressure, A1c), as well as inflammation (hsCRP) along with changes in microbial richness (Fig. 5).

Similar analyses looking for associations of relative changes in clinical parameters with changes in target genera (those showing significant changes one year after the interventions from univariate tests) indicated significant associations (FDR < 0.05) between changes in anthropometric variables, glucose sensitivity variables (glycated haemoglobin, insulin), and inflammatory variables (hsCRP), and also changes in Proteobacterial lineages, mostly of the gamma clade (Fig. 5 and Additional file 1: Figure S7). We found that improvement in anthropometric, metabolic and inflammatory profiles characterized by decrease in these variables after the interventions was associated with an increase in gamma-proteobacterial lineages, which were mostly driven by the RYGB surgery. Importantly, there was an inverse association between the concentration of *Klebsiella* and body weight, comparing the baseline to one year after follow-up of metabolic surgery, which corroborate the beneficial metabolic impact of the increase of gamma-proteobacteria after RYGB. Similarly, improvements in levels of HDL-C after intervention were positively associated with increases in these same gamma-proteobacterial clades, which was significant (FDR < 0.05) only for the *Enterobacter* genus. On the other hand, we found a positive association (although not significant) between changes in Firmicutes genera (*Ruminococcus*, *Faecalibacterium*) and changes in these same clinical variables explained by the phenotypic improvement, along with a decrease in these Firmicutes genera after one year of follow-up (Additional file 1: Figure S5).

## Discussion

Bariatric surgery treatment has increased worldwide, being the most efficient procedure for the treatment of severe obesity. The underlying beneficial metabolic effects go beyond weight loss, which has led to the consideration of RYGB for diabetic patients with milder forms of obesity. This is the first randomized controlled clinical trial that simultaneously explored clinical and microbiota changes in diabetic patients with class-1 obesity after RYGB *versus* standard medical therapy. We found that both RYGB and standard medical therapy groups improved anthropometric outcomes at 1 year of follow-up, with RYGB patients having significantly higher improvements. However, only RYGB patients achieved improvement/remission in diabetes status (n = 7, 87.5%) and, significantly improved anthropometric and glycaemic profiles, independently and progressively, during the first year of follow-up and simultaneously had gut microbiota changes. On the other hand, after an initial clinical improvement in medical therapy arm, at the final endpoint all participants failed to achieve diabetes remission or improvement. During the first 6 months the patients were observed 4 times (M0, M1, M3 and M6), which might contributed for the initial improvement seen in this arm, as support of healthcare professionals is one of the most important motivators for lifestyle changes and therapeutic adherence [31]. Sustainable progress after an intensive intervention is challenging and a gap of 6 months without clinical visits (between M6 and M12) might contributed for the decline on clinical benefits, which has also been supported by other authors [32, 33].

Gut microbiota plays a relevant role in the complex causes of obesity and T2D, and is hypothesised to be involved in the modulation of metabolic status after RYGB. Microbial richness is a simple descriptive parameter of the microbiome. A healthy microbial ecosystem is usually characterised by an elevated level of microbial richness [21, 22]. In addition, one of the key features of the microbiome that characterises obesity is a low level of microbial richness, which is correlated with metabolic disorders, such as low-grade inflammation, insulin resistance, and adipocyte size [21, 34]. Here, microbial richness was measured at the genus level. Despite a trend of lower baseline genus richness in patients in the surgical arm, we observed a continuous increase after RYGB during the 12 months of follow-up. Our results confirm previous observations that RYGB increases microbiome richness, not only in patients with morbid obesity, but also for a broader BMI range, including patients with a BMI 30–35 kg/m^2^ [29].

As expected, there was a strong inverse association between increase in microbial richness and improvement of clinical phenotype, including anthropometric, metabolic (waist circumference, diastolic blood pressure, A1c), and inflammatory (hsCRP) biomarkers. These results suggest that systemic and anatomical changes induced by RYGB can restore a putative loss of microbial richness with an improvement of metabolic profile. On the contrary, genus richness did not change in the standard medical therapy arm and ended-up being significantly lower at M12, with no differences in glycaemic profile, comparing to baseline. This suggests that standard medical therapy optimisation does not target the gut microbiota, which reinforces the hypothesis that modulation of gut microbiota by pre or probiotics could be a complementary strategy for improving glycaemic status in this context.

Across enterotypes, there were no significant differences in genus richness, nor in clinical variables at baseline. We hypothesise that as the sequencing depth was relatively low in this study, it provided a low number of observed genera, which significantly influenced the enterotyping outcome. Indeed, the evaluation of the Bray–Curtis distance is mostly driven by the most abundant genera, which affects the sensitivity of the analyses. Finally, the number of individuals in each group (four for the baseline K4 therapy, and five for the baseline K4 bypass) decreased the statistical power, making the interpretation difficult. However, we observe a significantly higher abundance of Bacteroides genus in the RYGB group in comparison with the medical group in baseline. This bacterial genus has been associated to a dysbiotic microbiome composition associated to low microbial diversity, low microbial cell density and enriched in several pathologies like Crohn disease, primary sclerosing cholangitis and Inflammatory Bowel Disease [24, 35]. This dysbiotic microbiome composition is also enriched in severe obese patients under RYGB before surgical intervention, decreasing progressively after bariatric surgery in parallel with improvements in microbial richness, clinical conditions and weight loss [36].

One year after surgery, we observed a significant decrease in three bacterial taxa belonging to the Firmicutes phylum (*Ruminococcus*, *Lachnospiraceae_unclassified* and *Faecalibacterium*), which are recognised as having anti-inflammatory properties with a beneficial impact on metabolic health [14, 29]. The decrease of Firmicutes lineages after bariatric surgery has been reported in different studies [37–39], whereas contradictory results are observed in different studies related to the presence of Faecalibacterium prausnitzii [30, 40, 41]. Some studies have also shown *Roseburia* inhibit pro-inflammatory cytokines (NF-kB) and was negatively associated with T2DM [42, 43]. In a recent study, *Roseburia* and *Lachnospiraceae* increase improved the likelihood of T2DM remission [44]. In our study, we did not find significant differences in Roseburia composition, though it was observed a *Lachnospiraceae_unclassified* decreased. This phenomenon might be associated with a diversity of genus inhabiting human gut, which turns strain-specific effects. Furthermore, it has been demonstrated that despite the beneficial effects of bariatric surgery on glycaemic control and weight reduction, compared to medical therapy alone, both HbA1c and body weight tend to increase over time [45]. This leads us to hypothesise that, despite RYGB improving the metabolic status in T2D patients with mild obesity, RYGB did not have the ability to restore a healthy microbiome composition, especially if it started with a highly dysbiotic microbiome state.

We also observed an increase in abundance of bacteria of the phylum Proteobacteria (*Klebsiella*, *Gammaproteobacteria*, *Enterobacter*, *Gammaproteobacteria_unclassified*), one year after the surgical intervention, as well as of *Veillonellaceae_unclassified* (Firmicutes phyla). Increases in Veillonella and other oral bacterial lineages after RYGB surgery has been reported in other studies associated to decrease of acid secretions consequence to the stomach size reduction, which could facilitate the intestinal colonization of oral bacteria [38, 41], whereas an increase in gamma-proteobacteria after bariatric surgery is a common finding—both in humans and in mice [30, 37, 46]. In our study, we also observed an increase of the Proteobacteria/Firmicutes ratio after RYGB. The increase of bacteria belonging to the phylum Proteobacteria was associated with the improvement of metabolic and inflammatory parameters after bariatric surgery [47]. In an animal model, the increase of proteobacteria was also accompanied by a reduction in inflammatory response and glucose homeostasis improvement [48]. The phylum Proteobacteria is composed of facultative anaerobes, and consequently oxygen increase [49] combined with higher pH after RYGB (in the gut) could contribute to an increase of these bacteria in parallel with improvements on metabolic health. If this increase in proteobacterial lineages after bariatric surgery have a direct contribution to improvement of health status of severe obese patients or is a response to the drastic anatomical changes in the gut environment consequence of the surgical procedure (high oxygen availability, higher pH, higher amounts of undigested nutrients in parallel to caloric restriction) would require further experiments with animal models in parallel with more precise characterization of enterobacterial lineages increased after bariatric surgery at strain level with shotgun metagenomics data and de-novo sequence assembly for better understanding of its functional role.

On the other hand, we did not observe any significant differences with regards to ratio Firmicutes/Bacteroidetes (rFB). The concept that obesity is associated with a lower percentage of Bacteroidetes and a higher percentage of Firmicutes in obese individuals is contradicted by several studies, which demonstrate that there is no difference in relative abundance of Firmicutes and/or Bacteroides and no association of weight loss with the rFB [50, 51] in obese individuals. Furthermore, T2D has been linked to a decreased abundance of Firmicutes and an increase in bacteria belonging to Bacteroidetes and Proteobacteria, when compared to obese patients [52]. However, it is difficult to validate these links from the results of our study, which included T2D patients with mild obesity, knowing the Firmicutes phylum and Bacteroides contain at least 250 and 20 genera, respectively. Higher taxonomic levels may not necessarily reflect specific bacteria changes, however, the results of our study are in line with those of Campisciano et al., as they corroborate that rFB is not a predictive biomarker of the outcome for metabolic surgery [53].

### Strengths and limitations

This study assessed patients’ evolution at different points in time over the first year of follow-up, including the first and third months after interventions, contrary to the majority of published studies which lack comprehensive data regarding the first six months following baseline and through a 12-month period. These intermediate timepoint assessments allowed us to monitor a clear evolution of the clinical profile.

The sample size could have impaired to a degree the multivariate analysis or the effect size of metabolic surgery outcomes, and differences in baseline characteristics can limit observations; however, the adjusted pairwise analysis performed can help to obviate these differences, as well as, the results throughout the timepoints were consistent with other studies. Of note is the fact that our observations can be limited by different technical aspects of microbiome analysis, including the collection, generation, and quantification of the abundance profiles. In addition, heterogeneity in dietary profiles and physical activity can also explain part of phenotypic outcomes during the treatment. Dietary data were mainly recorded for the medical arm, but not for the surgical arm throughout the different time-points, which limits our ability to control for food intake in the changes in microbiome profiles. Consequently, dietary analyses were not shown.

Currently anti-diabetic drugs have demonstrated to modulate and change gut microbiota and its metabolic capacity [54]. Conversely, gut microbiome can also influence drug metabolism and its effects [55]. Understanding the dynamics of drug-microbiome crosstalk would offer important insights for the development of personalized manipulation in the future, according to patients’ gut microbiota status. Unfortunately, our study was not designed to understand this bi-directional drug-microbiome interaction, which is a limitation of our study. Antibiotics also affect gut microbiota composition [56]. In this regard, we cannot exclude an influence of peri-operative antibiotics after bariatric surgery in gut microbiota changes, after metabolic surgery.

In conclusion, our research suggests that there is a remarkable phenotypic improvement after metabolic surgery which occurs simultaneously with gut microbiota changes. Nevertheless, gut microbiome changes alone cannot explain the beneficial metabolic health impact of RYGB. Other mechanisms such as diet, hormonal changes, bile acids metabolism, and physical activity need to be further explored in this equation in order to better explain the metabolic improvement of T2D patients with mild obesity after RYGB.

## Supplementary Information

**Additional file 1. **Additional tables and figures.

## Data Availability

The datasets used during the current study are available from corresponding author.

## Abbreviations

T2DM: Type 2 diabetes mellitus
RYGB: Roux-en-Y gastric bypass
BMI: Body mass index
RCT: Randomised controlled clinical trial
CHSJ: Centro Hospitalar São João
ISRCTN: International Standard Randomised Controlled Trial Number Registry
ADA: American Diabetes Association
TC: Total cholesterol
HDL-C: High-density lipoprotein cholesterol
TG: Triglycerides
LDL-C: Low-density lipoprotein cholesterol
PCoA: Principal Coordinate Analysis
DMM: Dirichlet Multinomial Mixture
SD: Standard deviation
HOMA-IR: Homeostasis model assessment of insulin resistance,
%WL: Percentage of total weight lost
