# Supplementary material for: Gut microbiota changes after metabolic surgery in adult diabetic patients with mild obesity: a randomised controlled trial
Source: Diabetol Metab Syndr. 2021 May 21;13:56. doi: 10.1186/s13098-021-00672-1 (PMC8139007; doi:10.1186/s13098-021-00672-1)
Supplement: Supplementary file 1 — Additional file 1. Additional tables and figures. [file 13098_2021_672_MOESM1_ESM.docx]

## Supplementary material

*Clinical changes during intervention*

Anthropometric and inflammatory measures were significantly reduced at any given endpoint in both arms (Supplemental Table S1 and Table S2). However, in the medical arm, differences between baseline and endpoints remained stable (with a BMI mean change from M0 to M1 and M12 in the medical arm: -0.98 kg/m^2^ and -1.52 kg/m^2^, respectively; a plasma hsCRP mean change from M0 to M1 and M12 in the medical arm: -1.25 mg/L and -1.22 mg/L, respectively), while in the surgical arm, the differences increased from one endpoint to another (BMI mean change from M0 to M1 and M12 in the surgical arm: -2.68 kg/m^2^ and -9.00 kg/m^2^, respectively; plasma hsCRP has a mean change from M0 to M1 and M12 in the surgical arm: -0.64 mg/L and -6.06 mg/L, respectively).

Metabolic profile was globally unchanged through endpoints in the medical arm (**Supplementary Table S1 and Table S2**). In the surgical arm, metabolic profile significantly improved at each timepoint (HbA_1c_ mean change from M0 to M1 and M12 in the surgical arm: -1.11% and -2.51%, respectively; with a mean change for triglycerides from M0 to M1 and M12 in the surgical arm: -10.00 mg/dL and -23.88 mg/dL, respectively).

Systolic and diastolic blood pressure levels were similar between the medical arm and the surgical arm at any given timepoint. However, only participants in thefrom surgical arm showed a significant reduction of systolic and diastolic blood pressure from M0 to M12 (systolic blood pressure mean change from M0 to M12 in the surgical arm: -12.50 mmHg; diastolic blood pressure mean change from M0 to M12 in the surgical arm: -7.38 mmHg). The linear regression analysis focused on the surgical arm only, where we found no association between HbA_1c_ and BMI at any given time point (at M1: r^2^ 0.019, p=0.745; at M12: r^2^ 0.013, p=0.786).

## Supplementary Tables

**Table S1.** **Mean changes between baseline and time points (1, 3, 6, and 12 months of follow-up) in the medical arm.**

|  | **Month 1**  **(mean change)** | **p** | **q** | **Month 3**  **(mean change)** | **P** | **q** | **Month 6**  **(mean change)** | **p** | **q** | **Month 12**  **(mean change)** | **p** | **q** |
| --- | --- | --- | --- | --- | --- | --- | --- | --- | --- | --- | --- | --- |
| **Body weight (kg)** | -2.67 | **0.005** | 1.18 | -4.66 | **0.001** | 1.54 | -4.40 | **0.005*** | 1.15 | -4.19 | **0.022*** | 0.88 |
| **BMI (kg/m^2^)** | -0.98 | **0.004** | 1.22 | -1.70 | **0.001** | 1.63 | -1.60 | **0.005*** | 1.18 | -1.52 | **0.022*** | 0.88 |
| **%WL (%)** | -3.15 | **0.003** | 1.26 | -5.49 | **<0.001** | 1.68 | -5.18 | **0.004** | 1.21 | -4.89 | **0.021** | 0.88 |
| **Waist circumference (cm)** | -3.75 | **<0.001** | 1.91 | -5.00 | **0.001** | 1.58 | -7.49 | **0.002*** | 1.39 | -7.41 | **0.001*** | 1.54 |
| **Visceral fat area (cm^2^)** | -6.77 | **0.003** | 1.31 | -11.07 | **0.002** | 1.39 | -8.34 | **0.047*** | 0.73 | -8.83 | **0.042*** | 0.75 |
| **Body fat mass (kg)** | -2.08 | **<0.001** | 2.06 | -3.35 | **<0.001** | 1.81 | -2.01 | 0.112 | 0.56 | -2.80 | **0.018*** | 0.91 |
| **Fat-free mass (kg)** | -0.60 | 0.221 | 0.42 | -1.32 | 0.055 | 0.70 | -1.50 | 0.073 | 0.64 | -1.32 | 0.123 | 0.54 |
| **Fasting glucose (mg/L)** | -20.40 | 0.128 | 0.53 | -20.20 | 0.224 | 0.41 | -5.60 | 0.661 | 0.14 | -5.20 | 0.725 | 0.11 |
| **HbA_1c_ (%)** | -0.74 | **0.001** | 1.46 | -1.14 | **0.009** | 1.06 | -0.92 | **0.028*** | 0.82 | -0.45 | 0.114 | 0.55 |
| **Insulin (⎧U/mL)** | 2.16 | 0.181 | 0.46 | 0.86 | 0.756 | 0.10 | 2.38 | 0.196 | 0.43 | 2.59 | 0.458 | 0.25 |
| **C-peptide (ng/mL)** | 0.29 | 0.333 | 0.32 | -0.46 | 0.187 | 0.45 | -0.35 | 0.253 | 0.39 | -0.24 | 0.594 | 0.17 |
| **HOMA-IR** | -0.06 | 0.938 | 0.03 | -0.46 | 0.616 | 0.16 | 0.63 | 0.386 | 0.29 | 2.02 | 0.373 | 0.30 |
| **Total cholesterol (mg/dL)** | -11.80 | 0.162 | 0.48 | -19.50 | 0.291 | 0.35 | -5.50 | 0.642 | 0.15 | -10.10 | 0.421 | 0.27 |
| **Triglycerides (mg/dl)** | -4.00 | 0.754 | 0.10 | -9.90 | 0.836 | 0.07 | -42.50 | 0.401 | 0.28 | -17.30 | 0.564 | 0.19 |
| **HDL-c (mg/dL)** | 0.80 | 0.665 | 0.14 | 0.30 | 0.855 | 0.06 | 1.00 | 0.409 | 0.27 | 1.80 | 0.347 | 0.31 |
| **LDL-c (mg/dL)** | -10.80 | 0.145 | 0.50 | -27.30 | 0.100 | 0.58 | -7.50 | 0.551 | 0.20 | -18.00 | 0.216 | 0.42 |
| **Systolic BP (mg/dL)** | -10.70 | **0.039** | 0.76 | -15.30 | **0.030** | 0.81 | -8.00 | 0.088 | 0.61 | -7.70 | 0.192 | 0.45 |
| **Diastolic BP (mg/dL)** | -0.10 | 0.980 | 0.01 | -0.90 | 0.804 | 0.08 | 1.40 | 0.665 | 0.14 | 0.20 | 0.968 | 0.01 |
| **hsCRP (mg/L)** | -1.25 | **0.021** | 0.89 | -1.18 | **0.010** | 1.02 | -1.31 | **0.038*** | 0.77 | -1.22 | **0.035*** | 0.78 |

BMI: body mass index; %WL: percentage of weight lost; HbA_1c_: Glycated hemoglobin; HOMA-IR: homeostatic model assessment for insulin resistance; HDL-c: high density lipoprotein cholesterol; LDL-c: low density lipoprotein cholesterol; BP: blood pressure; hsCRP: high sensitivity C-reactive protein; n.a.: not applicable.

Bold values are statistically significant.

**Table S2. Mean changes between baseline and time points (1, 3, 6, and 12 months of follow-up) in the surgical arm.**

|  | **Month 1**  **(mean change)** | **p** | **q** | **Month 3**  **(mean change)** | **p** | **q** | **Month 6**  **(mean change)** | **p** | **q** | **Month 12**  **(mean change)** | **p** | **q** |
| --- | --- | --- | --- | --- | --- | --- | --- | --- | --- | --- | --- | --- |
| **Body weight (kg)** | -9.85 | **<0.001** | 2.92 | -16.41 | **<0.001** | 4.71 | -21.48 | **<0.001*** | 5.55 | -23.29 | **<0.001*** | 3.22 |
| **BMI (kg/m^2^)** | -2.69 | **0.040** | 0.89 | -5.96 | **<0.001** | 5.08 | -7.90 | **<0.001*** | 4.41 | -9.00 | **<0.001*** | 2.50 |
| **%WL (%)** | -10.46 | **<0.001** | 3.71 | -17.67 | **<0.001** | 3.60 | -23.37 | **<0.001*** | 5.06 | -25.49 | **<0.001*** | 2.98 |
| **Waist circumference (cm)** | -8.81 | **0.004** | 1.50 | -15.00 | **<0.001** | 2.93 | -19.81 | **<0.001*** | 5.43 | -24.64 | **<0.001*** | 3.29 |
| **Visceral fat area (cm^2^)** | -17.23 | **0.001** | 2.11 | -40.89 | **<0.001** | 3.27 | -65.29 | **<0.001*** | 3.82 | -72.61 | **<0.001*** | 3.86 |
| **Body fat mass (kg)** | -6.33 | **<0.001** | 2.54 | -13.55 | **<0.001** | 4,44 | -20.06 | **<0.001*** | 5.89 | -21.98 | **<0.001*** | 3.52 |
| **Fat-free mass (kg)** | -3.51 | **<0.001** | 2.56 | -2.86 | **0.009** | 1.27 | -1.41 | 0.081 | 0.72 | -1.31 | 0.171 | 0.54 |
| **Fasting glucose (mg/L)** | -21.50 | 0.280 | 0.41 | -46.88 | **0.004** | 1.49 | -57.88 | **0.001*** | 2.12 | -51.00 | **0.001*** | 1.96 |
| **HbA_1c_ (%)** | -1.11 | **0.007** | 1.35 | -1.95 | **0.002** | 1.71 | -2.45 | **<0.001*** | 2.45 | -2.51 | **<0.001*** | 2.22 |
| **Insulinemia (⎧U/mL)** | -6.83 | 0.090 | 0.76 | -7.71 | 0.043 | 0.87 | -8.06 | **0.042*** | 0.88 | -9.01 | **0.014*** | 1.15 |
| **C-peptide (ng/mL)** | -0.19 | 0.553 | 0.22 | -0.50 | 0.166 | 0.55 | -0.71 | **0.043*** | 0.88 | -0.84 | **0.019*** | 1.07 |
| **HOMA-IR** | -3.53 | 0.100 | 0.73 | -4.09 | **0.035** | 0.92 | -4.40 | **0.025*** | 1.01 | -4.56 | **0.013*** | 1.15 |
| **Total cholesterol (mg/dL)** | -10.13 | 0.465 | 0.27 | -3.13 | 0.784 | 0.10 | -7.88 | 0.446 | 0.29 | 2.63 | 0.758 | 0.11 |
| **Triglycerides (mg/dl)** | -10.00 | 0.392 | 0.32 | -16.25 | 0.168 | 0.54 | -32.50 | **0.011*** | 1.22 | -23.88 | **0.004*** | 1.46 |
| **HDL-c (mg/dL)** | -4.63 | 0.247 | 0.45 | -1.00 | 0.692 | 0.15 | 2.38 | 0.514 | 0.24 | 10.88 | **0.010*** | 1.23 |
| **LDL-c (mg/dL)** | -2.50 | 0.857 | 0.07 | 1.13 | 0.921 | 0.04 | -3.75 | 0.694 | 0.15 | -3.63 | 0.666 | 0.16 |
| **Systolic BP (mg/dL)** | -15.25 | **0.022** | 1.03 | -10.75 | 0.073 | 0.75 | -13.25 | **0.002*** | 1.73 | -12.50 | **<0.001*** | 2.30 |
| **Diastolic BP (mg/dL)** | -7.88 | **0.008** | 1.31 | -5.00 | 0.068 | 0.76 | -5.00 | **0.010*** | 1.25 | -7.38 | **0.005*** | 1.45 |
| **hsCRP (mg/L)** | -0.64 | 0.848 | 0.07 | -4.95 | **0.001** | 1.91 | -5.76 | **<0.001*** | 2.41 | -6.06 | **<0.001*** | 2.49 |

BMI: body mass index; %WL: percentage of weight lost; HbA_1c_: Glycated hemoglobin; HOMA-IR: homeostatic model assessment for insulin resistance; HDL-c: high density lipoprotein cholesterol; LDL-c: low density lipoprotein cholesterol; BP: blood pressure; hsCRP: high sensitivity C-reactive protein; n.a.: not applicable.

Bold values are statistically significant.

**Table S3.** **Microbial richness relationship with clinical phenotypes**

**Table S4. Differentially abundant genera in each group**

## Supplementary Figures

***
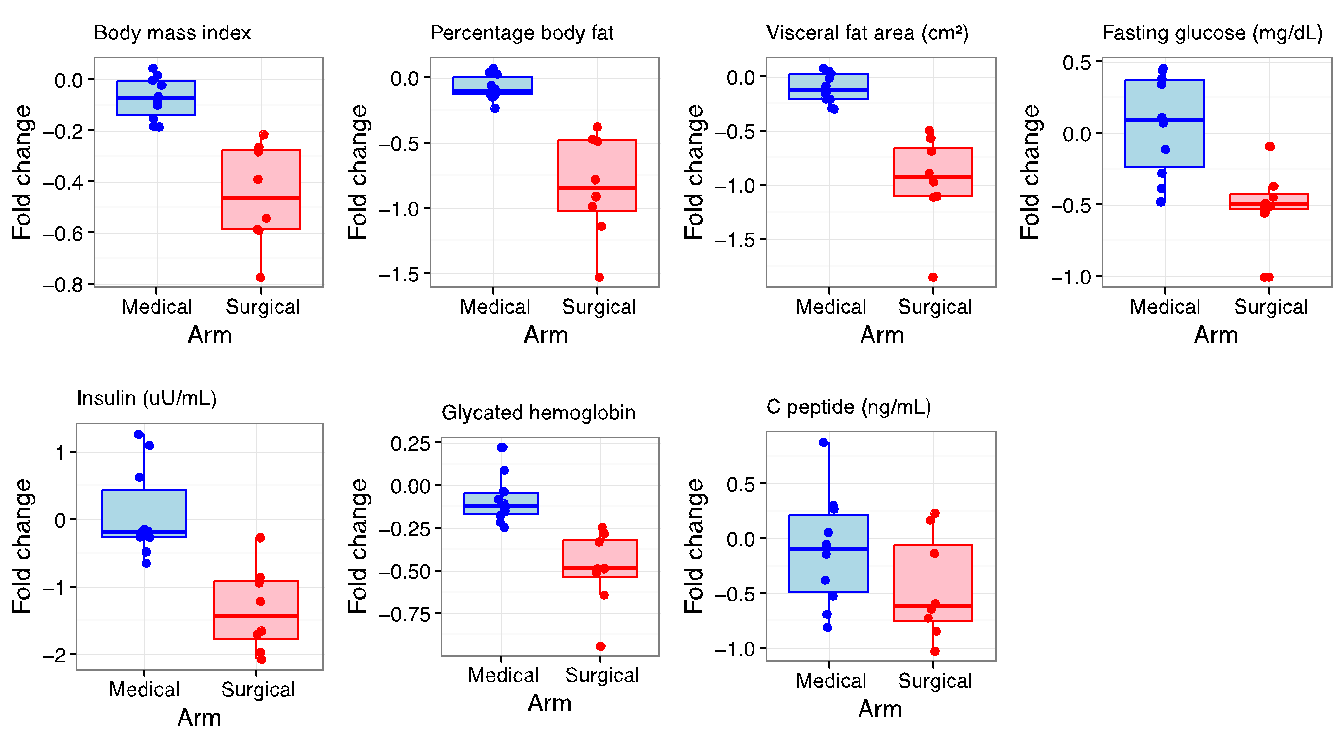
***

**Figure S1. Fold change computed as value at (M12-M0)/M0 is presented for the clinical variables above.**

We can easily notice a greater improvement in percentage in the RYGB arm.

*Characterisation of the gut microbiome*

*
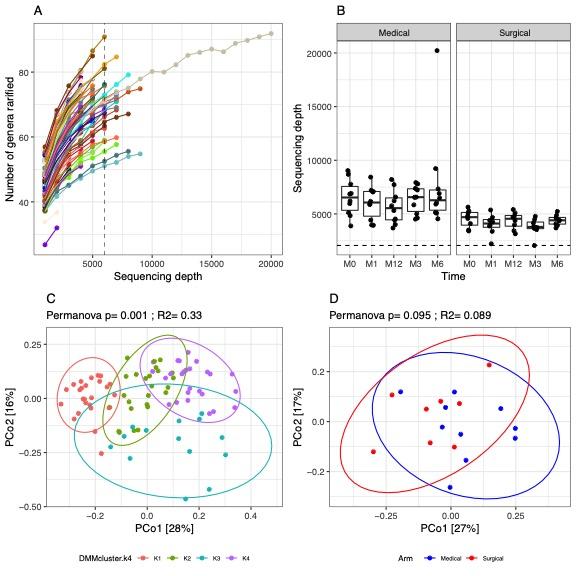
*

**Figure S2. rarefaction analyses, enterotypes in DMM k-4**

**A**: Genus richness as a function of rarefaction for each sample The threshold of 6000 reads is depicted by the vertical dashed line. **B**: Boxplots of sequencing depth by time point and study group. We notice an overall lower depth in the surgical group. **C**: Principal Coordinates Analyses of all samples coloured by enteroytpe classification using DMM approach with k=4. Permanova test was used to evaluate the impact of enterotype classification in microbiome composition. **D**: Principal Coordinates Analyses of baseline samples coloured by clinical group. Non-significant differences were observed in microbiome composition between both clinical arms (medical, surgical) according with Permanova test.


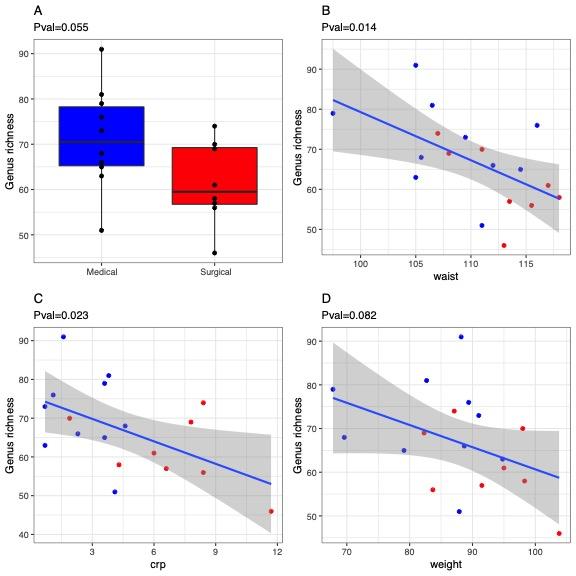


**Figure S3. Microbial richness at baseline and relationship with clinical phenotypes.**

(A): genus richness distribution at baseline by study group. (B-D): scatter-plots between significant clinical phenotypes and genus richness (p-value in Spearman correlations<0.1).

***
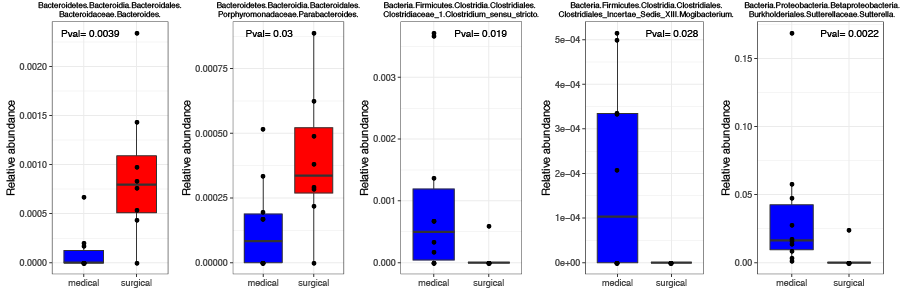
***

***Figure S4. Microbial genus with significant differences between both study groups. P-values comes from non-parametric Wilcoxon tests. None of them resist P-value adjustment for multiple comparisons (Benjamini-Hochberg method).***


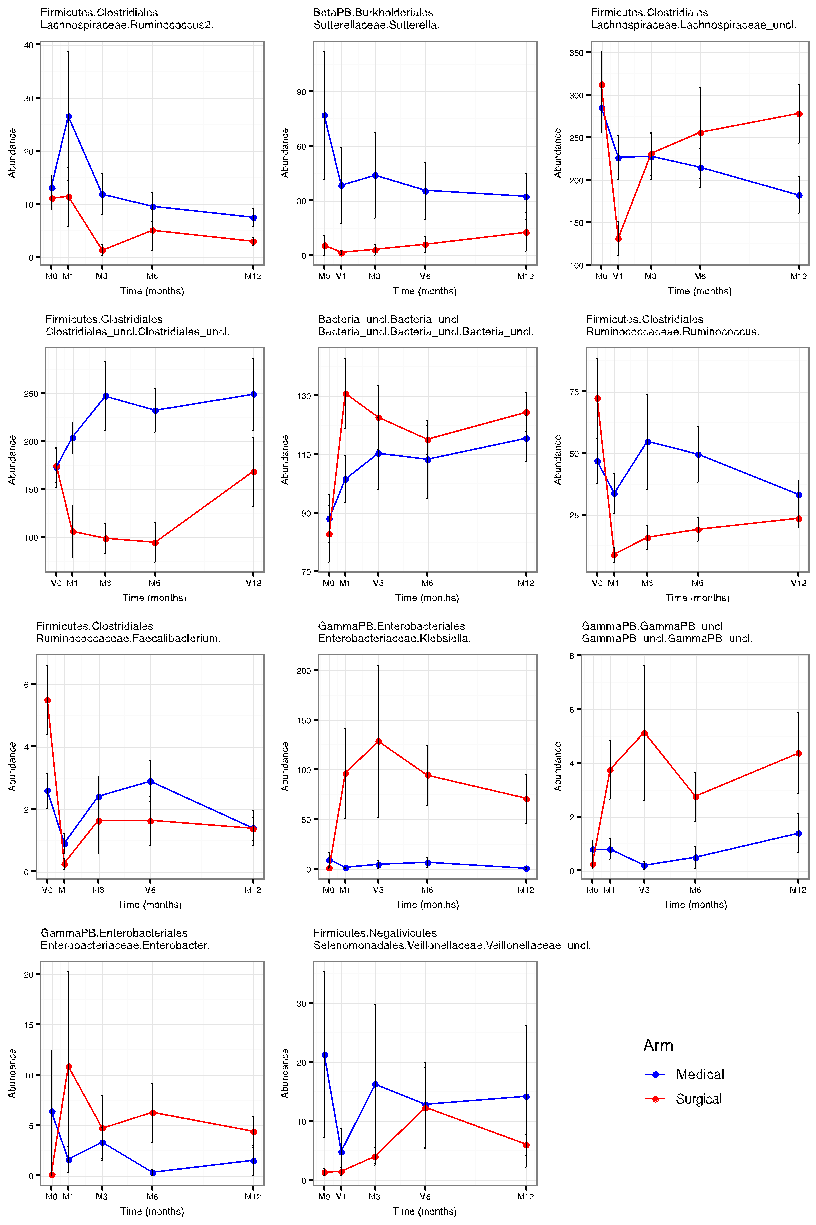


**Figure S5. Significant fold changes in genera profiles by time and by study arms.**

Microbial genus with significant differences in abundance distributions between baseline and month 12 in medical or surgical arms (pvalue Paired Wilcoxon tests <0.05; Table S5).


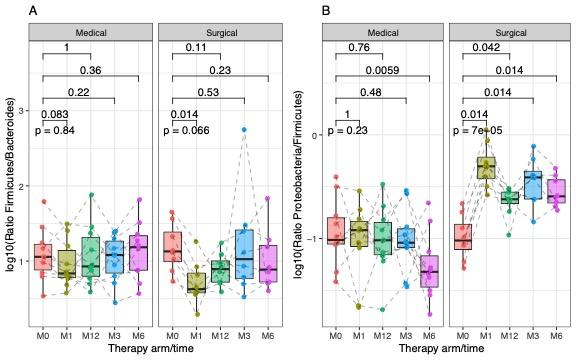


**Figure S6**. **Ratio marker evolution through time and study arms.**

**A:** Boxplots indiciating the Firmicutes/Bacteroidetes ratio across time and study arm **B:** Boxplots indiciating the Proteobacteria/Firmicutes ratio across time and study arms. P-values of non-parametric paired Wilcoxon tests between baseline and other time points are indicated.


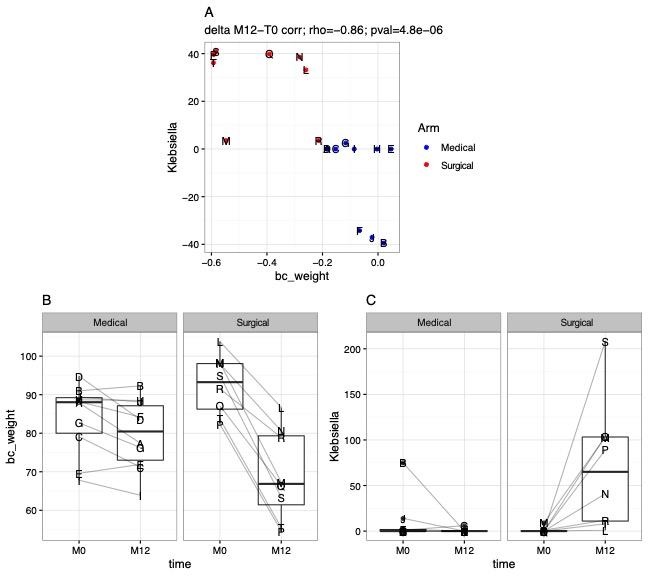


**Figure S7: Association between changes in weight and changes in *Klebsiella* genus abundance after interventions**

**A**: Correlation of relative changes in Klebsiella genus abundance and changes in Weight between baseline and month 12. **B**: Boxplots illustrating distribution of weight and **C**: boxplot indicating distribution of Klebsiella genus abundance between baseline (M0) and month 12 (M12) stratified by study arm.
